# Three Cases of Gun-Shot Wound of Bladder

**Published:** 1864-07

**Authors:** W. H. Butler

**Affiliations:** Acting Assistant Surgeon, U. S. A.


ART. II.—*Three Cases of Gun-Shot Wound of Bladder. By the late* W. H. BUTLER, *Acting Assistant Surgeon, U. S. A.*

*Fibrinous Thickening of Bladder.*—Charles Wolver, aged 20, private Co. "H," 24th New York Volunteers, was wounded at Centreville, August 30th, 1862, by a musket ball entering above pubis and one inch to right of median line, passing obliquely to the left and downward, coming out four nches above coccyx and three inches to the left of median line. Entered hospital September 1st. Posterior wound closed when he entered the house. From the anterior wound urine has almost constantly flowed, although a flexible catheter has been placed in the urethra from the beginning. Pulse

125. Difficulty has been experienced in passing the catheter, which was eventually overcome by withdrawing the stylet gradually after the catheter passed under the pubis, giving it an upward tilt on the inner end. Pulse 110 to 125 most of the time since. Treatment—cleanliness, good diet, beef tea, meat broth, etc.; opium in full doses. Bowels confined until September 9th, when the tongue became thickened and coated, and he complained of oppression in his bowels. ℞ Enema warm water; a moderate evacuation followed; no pus or blood.

10th.—Has marked jaundice. ℞ Mass hydrag. gr. vi.; follow in six hours oleum ricini ℥ i. Had a free and painless evacuation.

11th.—Vinum album, ovum vitellus No. 1, every 4 hours; pulv. ipec. comp. gr. xij. S. quinia gr. xii, mass hydrag. gr. vij. bed time; was quiet, until the latter part of the night, when he became restless and delirious.

12th.—Pulse 120 and weaker. ℞ Milk punch, ℥ iv. every 1½ hours beef essence, etc. 6 P. M. ℞ Tinct. opii. ʒ iv. spts. vini gallici ℥ i. sig. 1 teaspoonful every hour till he sleeps. Spits up clots of blood to-night, and has some through the day; respirations accelerated, pulse 140; he gradually failed, and died 8 A. M., September 13th, living 14 days with this serious wound, which seemed almost hopeless from the beginning. The jaundice seemed to be almost immediately developed after the first evacuation of the bowels. This might have been accidental, and probably was.

Post mortem 3 hours after death by Drs. Butler and Kennon, Mr. Fuller present. Appearance of body very yellow and emaciated. Rigor mortis well marked, bowels tympanic, eyes sunken, sugillation well marked on the back, neck and thighs, Pupils markedly dilated; external appearance of wound very dark and dry. Found on cutting through abdominal walls slight fracture of superior part of pubis, some small pieces of bone being detached. The ball entering on the upper and to the right side of the bladder, ulceration had taken place to considerable extent beneath the parieties of abdomen and to the right of bladder. A small piece of bone was driven into the bladder from the os pubis. Walls of the bladder thickened at least an inch and its capacity lessened, say one half. The ball passed through the left side of bladder and through upper part of ischiatic notch. Outer wound completely closed, and could not be forced open. Lungs normal anteriorly; left lobes covered with plastic lymph, and cavity of chest filled with serum, (of a pale yellow color,) and the Pericardium also filled with serum. Liver much enlarged, estimated to weigh six pounds; kidneys normal; spleen congested. Heart normal externally; on opening the cavities, found them all filled with thick and tenacious fibrinous bands; this was particularly marked in the right auricle, the mass on being removed taking casts of the veins well up into the head; walls of usual thickness. Some clotted blood on left ventricle, mixed with the fibrine or lying over it; stomach and intestines healthy.

*Gun-shot Wound through Bladder.*—John Mahay, age 19, private Co. H, 101st N. Y. Vols., was wounded at the battle of Bull Run, August 29, 1862, the ball entering the crest of the pubis about an inch to the right of the symphysis and passing directly through the bladder in a downward and outward direction, making its exit between the spine of ischium and coccyx. Several pieces of bone have at different periods passed through the urethra, and although he has never been perfectly free from pain, sometimes of the most severe character, his appetite and strength continued remarkably good up to a late period.

The wounds made by the entrance and exit of the ball would close up for a longer or shorter period and again open and discharge urine, pus and blood; and when urinating that fluid would pass quite as freely through these fistulas as through the urethra. Generally urinated freely, but never without pain referred to the penis and perineum, at times very severe, the urine always albuminous, muco-purulent or bloody, and in considerable quantities.

During the early part of the treatment, a catheter was retained in the bladder, and the attempt has been made at different periods to introduce it, but was attended with so much suffering that the patient was unable to endure it; it never seemed to be of much benefit. Has suffered severe pain referred to the kidneys at different periods, which was allayed by cupping, warm fomentations and opiates.

About six weeks ago he was placed under the influence of ether, and the anterior opening dilated, through which an irregular shaped piece of bone was extracted, and at the same time a stone was distinctly felt, but it was not deemed prudent to operate for its removal at that time, since which time, however, he has been gradually failing, and died on the evening of the 24th instant. Died Oct. 24/63

Autopsy the following day, when it was discovered that the course of the ball varied but little from the foregoing description; the bladder greatly contracted, and the walls or coats three-eighths of an inch thick, and its cavity nearly filled by two stones weighing ʒ ij. grs. x, and ʒ ij. grs. lvi.

=ʒ vi. gr. vii.; several pieces of necrosed bone removed from point of exit of ball.

Sherman E. Perry, age 27, private Co. "K," 16th N. Y. Vols. Wounded at the battle of Salem Church, near Fredericksburgh, Va., late in the afternoon of May 3d, 1863, body inclined forward when he was struck by a conical ball, which first passed through his canteen and then entered the body about half way between the anterior superior spinous process of ilium and the symphasis pubis, and two inches below a line drawn from one anterior superior spine of the ilium to the other at a point corresponding to the inguinal canal, passing downwards, backwards and to the right, through the right side of scral bone, and making its exit midway between the sacro-coccygeal articulation and the great trochanter of the right femur. He fell to the ground when struck, but soon got up and walked some 40 rods to the rear, where he entered a small house. He lost considerable blood the first twenty-four hours, and thinks the bladder was about half full when the ball entered, judging from the time he last urinated. I ought to have said that the ball lodged at a point above described as the exit, from which spot it was extracted on the 4th day by a surgeon of the 121st New York, who attended to the case until the 12th, at which time he was taken by the rebels to United States Ford and delivered up to our officers, taken across on a pontoon, and then carried in an ambulance to Potomac Creek Hospital, and attended to by Surgeon Oakly, of the 6th army corps. No urine was passed through the urethra for eight days, but blood and urine passed freely through the wound. He remained here until the 13th day of June, when he was transferred and admitted into Armory Square Hospital, by which time the wound had nearly healed up. A flexible catheter was constantly retained in the bladder for about four weeks previous to his admission, and continued for three or four days afterwards, about which time, on withdrawing the catheter, a piece of blue cloth immediately followed, which was rolled upon itself and was being very nicely encrusted with fine sand, serving as the nucleus for the formation of a stone. On the 21st of June, and after the introduction of a catheter, a small, flat piece of bone passed through the uretha. It was well known that something still remained in the bladder, from the fact of his having pain and difficulty in urinating, and at times the urine would suddenly cease to flow, which condition of things continued until the 21st of July, when he experienced unusual pain in attempting to urinate, and the cause soon became apparent in the shape of a stone, measuring about three-fourths of an inch long and one-half an inch in diameter, which resembles a peanut more than anything else in size, shape and color. He suffered very severe pain during its passage to the end of penis, from which place it was extracted with small forceps. Sept. 9th.—The evidence of further deposits in the bladder being conclusive and giving the patient trouble, Dr. D. W. Bliss, surgeon in charge, performed the lateral operation for stone, and removed a soft calculus of a flat oval in shape, three-fourths of an inch long, one-half inch wide and one-fourth inch thickness, the nucleus of which seemed to be cloth; weight 23 grains.

Patient made a rapid and complete recovery, and was transferred to New York Oct. 28, 1863, about which time his term of service expired.

